# The Role of Digital Technologies in Responding to the Grand Challenges of the Natural Environment: The Windermere Accord

**DOI:** 10.1016/j.patter.2020.100156

**Published:** 2021-01-08

**Authors:** Gordon S. Blair, Richard Bassett, Lucy Bastin, Lindsay Beevers, Maribel Isabel Borrajo, Mike Brown, Sarah L. Dance, Ada Dionescu, Liz Edwards, Maria Angela Ferrario, Rob Fraser, Harriet Fraser, Simon Gardner, Peter Henrys, Tony Hey, Stuart Homann, Chantal Huijbers, James Hutchison, Phil Jonathan, Rob Lamb, Sophie Laurie, Amber Leeson, David Leslie, Malcolm McMillan, Vatsala Nundloll, Oluwole Oyebamiji, Jordan Phillipson, Vicky Pope, Rachel Prudden, Stefan Reis, Maria Salama, Faiza Samreen, Dino Sejdinovic, Will Simm, Roger Street, Lauren Thornton, Ross Towe, Joshua Vande Hey, Massimo Vieno, Joanne Waller, John Watkins

**Affiliations:** 1 Lancaster University, Lancaster, UK; 2 Aston University, Birmingham, UK; 3 Joint Research Centre (JRC), European Commission, Ispra, Italy; 4 Institute for Infrastructure and Environment, Heriot-Watt University, Edinburgh, UK; 5 UK Centre for Ecology & Hydrology (UKCEH), Bangor, UK; 6 University of Reading, Reading, UK; 7 Telecom Paris, LTCI, IPParis, Paris, France; 8 Somewhere-nowhere, The Lake District, Cumbria, UK; 9 Natural Environment Research Council (NERC), Swindon, UK; 10 Science and Technology Research Council (STFC), Swindon, UK; 11 Environment Agency, Rotherham, UK; 12 Griffith University, Southport, QLD, Australia; 13 Joint Nature Conservation Committee (JNCC), Peterborough, UK; 14 JBA Trust, Skipton, UK; 15 STeAPP, UCL, London, UK; 16 Informatics Lab, Met Office, Exeter, UK; 17 Medical School, University of Exeter, Exeter, UK; 18 University of Oxford, Oxford, UK; 19 Alan Turing Institute, London, UK; 20 University of Leicester, Leicester, UK

**Keywords:** digital technologies, digital environment, data science, environmental science

## Abstract

Digital technology is having a major impact on many areas of society, and there is equal opportunity for impact on science. This is particularly true in the environmental sciences as we seek to understand the complexities of the natural environment under climate change. This perspective presents the outcomes of a summit in this area, a unique cross-disciplinary gathering bringing together environmental scientists, data scientists, computer scientists, social scientists, and representatives of the creative arts. The key output of this workshop is an agreed vision in the form of a framework and associated roadmap, captured in the Windermere Accord. This accord envisions a new kind of environmental science underpinned by unprecedented amounts of data, with technological advances leading to breakthroughs in taming uncertainty and complexity, and also supporting openness, transparency, and reproducibility in science. The perspective also includes a call to build an international community working in this important area.

## The Bigger Picture

Digital technology is having a major impact on many areas of society, and there is equal opportunity for impact on science in addressing grand scientific challenges. This is particularly true in the environmental sciences as we seek to understand the complexities of the natural environment under climate change. This perspective reports on the outcomes from a summit in this area, attended by 42 researchers selected as leading experts operating at the interface between digital technology and the environmental sciences. The key output of this workshop was the Windermere Accord, a collective statement around what is required to achieve a transformative effect through digital technology based around four key pillars of investigation, namely using technology to tame uncertainty; growing advocates and champions to enable, empower, and influence; embracing a new open and transparent style of science; and enabling integration and sophisticated treatment of feedbacks in complex environmental systems. These pillars all feed into the decision-making processes and are supported by a growing community. Looking forward, the accord also identified a pathway with particular emphasis on building an international, cross-disciplinary community to address the key challenges and achieve the real opportunities around digital technology and the environment.

## Teaser

This perspective presents the outputs of a summit in the area of how digital technology can help us respond to the grand challenges of environmental change. The resultant Windermere Accord contains a framework and associated roadmap, envisioning a new kind of environmental science underpinned by unprecedented amounts of data, with technological advances leading to breakthroughs in taming uncertainty and complexity, and also supporting openness, transparency, and reproducibility in science.

## Introduction

Digital technology is having a major impact on many areas of society, stimulating innovations in areas as diverse as smart cities, healthcare, energy (smart grid), and logistics. For this paper, we define digital technology as “the branch of scientific or engineering knowledge that deals with the creation and practical use of digital or computerized devices, methods, systems, etc.”^1^ Digital technology also has the potential to revolutionize the way we carry out science and address grand scientific challenges. This is certainly true in the environmental sciences, where new tools can both deepen our understanding of the natural environment and help determine well-founded mitigation and adaptation strategies and policies in the face of environmental change.

This short paper reports on the findings of a summit examining the role of digital technology in responding to the grand challenges of environmental change. This summit was held in the Lake District, UK, on 10–12 October, 2018, and represented a unique cross-disciplinary gathering bringing together leading researchers working at the interface between digital technology and environmental science with a view of exploring the potential contributions of digital technology in addressing the pressing issues around the natural environment. The summit used a process of creative facilitation to encourage the necessary cross-disciplinary conversation and to achieve our goals.

The paper discusses in particular the shared vision in the form of a framework and roadmap produced at the event, which we collectively refer to as the Windermere Accord, and issues a call to build the international community necessary to achieve this vision. The paper starts with background and context for the event and the organization of the summit and methods employed in reaching our consensus, leading up to a description of the accord. We also include a retrospective on how things have developed since.

## Summit: Background and Context

### Digital Technology

Digital technology is a fast-moving field that, as mentioned earlier, is having a profound impact on the way we live. We focus on several areas of innovation that have the most potential to be transformative on the environmental sciences:

1 The ability to acquire unprecedented amounts of environmental data: utilizing technologies such as remote sensing, cheap and ubiquitous sensing devices, and, more generally, the Internet of Things, citizen science, and additional data mined from the Web^2^
2 The ability to store and process big data through the massive and elastic/on-demand resources offered by cloud computing^3^
3 The ability to make sense of these big data and extract meaningful patterns through breakthroughs in data science and artificial intelligence (AI), thus generating new scientific knowledge, particularly when combined with process understanding from the environmental sciences^2^^,^^4^^,^^5^
4 The ability to visualize, present, and interact with these data and their subsequent analyses to support communication to different stakeholder groups, and hence support informed decision making

We note as well that this supports a chain of innovation affecting all aspects of the scientific process from data acquisition, through storage and processing and subsequent analyses, to communicating and collaborating over the results. We also note that, alongside the profound positive impact of such technologies, there is also a significant risk that they can have negative impacts on society, including through their greenhouse gas emissions,^6^ and it was important to acknowledge this and take it into account in the summit.

### Grand Challenges of Environmental Science

The environmental sciences are also going through an important transition toward a scientific discourse that is responding to:

1 The unpreceded amounts of environmental data related to different environmental facets, at different locations and scales^2^
2 The need to move toward a more open, cross-disciplinary, and collaborative style of science^7^ as demanded by the grand challenges of the natural environment; e.g., addressing food security, climate change, clean air/water
3 The need to embrace FAIR ( findable, accessible, interoperable, and reusable) principles in managing and accessing environmental data^8^^,^^9^
4 The need for a more holistic approach based on systems thinking to address the complexities of environmental ecosystems and their interactions
5 The subsequent need to integrate data and models to answer scientific questions around (complex) ecosystems

### A Digital Environment

It is interesting to note that there is a strong relationship between the changing nature of the environmental sciences and the areas of digital innovation identified earlier. Because of this, there is significant interest in what some observers call a digital environment; i.e., seeking ways in which digital technology can support a deeper understanding of the natural environment. In the UK, UK Research & Innovation (UKRI) has recently announced an ambitious cross-research council Strategic Priority Fund with a Constructing a Digital Environment (CDE) program.^10^ In their call document, they state:

By harnessing […] advances in technology […], there is an opportunity to create a digitally enabled environment [that] will deliver the capacity to improve the understanding and modelling of longer term environmental change and the prediction of events.

Similarly, Microsoft have recently launched a $50 million program, AI for Earth, looking at the potential transformative power of AI/data science coupled with cloud technology and how it can help society to step toward more sustainable solutions in for key areas, namely climate, water, agriculture, and biodiversity.^11^ Google have launched a sustainability mission building environmental sustainability “into everything they do.”^12^ There are also various other small to medium-sized communities emerging on around this theme; e.g., in climate informatics^13^, the Information and Communications Technology for Sustainability (ICT4S) community and conference series,^14^ sustainability informatics,^15^ IS-GEO,^16^ and Modeling for Sustainability.^17^

Although efforts are somewhat fragmented, all agree that the digital environment is fundamentally a cross-disciplinary area of study requiring collaboration between environmental scientists, computer scientists, data scientists, social scientists, and creative disciplines working closely together to address the role of digital technology in this important area.

## Summit: Organization

### Goals of the Summit

The goals of the summit were as follows:

• To provide a timely forum for the necessary dialogue between those working at the cutting edge of technology and those working on grand challenges of the natural environment
• To establish a shared vision and roadmap of what is required to allow the potential of digital technologies to be realized in this area
• To build an international community working on the resultant open research questions

### Process and Methodology

The summit was attended by 42 researchers (who are also co-authors of this paper), selected as leading experts operating at the interface between digital technology and the environmental sciences. The Ensemble research team^18^ hosted the summit in support of their vision of working together for digitally inspired integrated environmental science.

The participants were selected to achieve a balanced representation across the different underlying disciplines of the environmental sciences, computer science, and data science with representation from creative disciplines and social sciences. We also sought to ensure good and balanced coverage of (1) the chain of innovation from data acquisition through to support for decision making, (2) the different challenges being faced by environmental sciences as they address global challenges related to environmental change, (3) representatives of the emerging digital environment community, including research councils.

The methodology adopted in the workshop was one of creative facilitation to achieve the necessary cross-disciplinary discussion. This involved bespoke activities, stepping through a variety of phases and involving small/medium-sized and whole-group discussions, provocations, select presentations, pitches, and panel discussions that were designed to move the participants through key thresholds by eliciting responses to the following questions:

• What motivated you to be here, and what do you want to get out of the event?
• What are research challenges and opportunities around the digital environment?
• How ambitious could and should this community be?
• What are the barriers and obstacles to achieving this and (later) how can they be overcome?
• What should the main research foci be of this community?
• What mechanisms would allow us to drive this forward?
• What must we not lose sight of as we leave this summit?

The groups were constantly changed to maximize interaction across the set of participants, and outputs from one discussion were often used as inputs to future discussions to encourage ideas to percolate through the collective group.

### Facilitated Discussion: From Motivation to Consensus

The process involved a number of phases inspired by the methodology and questions introduced earlier in this article.

The first phase involved everyone capturing their motivations for attending the workshop followed by three rounds of trialogues (i.e., three-way conversations) based on these motivating statements. This session was important in establishing the participatory approach and giving people time to get to know each other and set out what they wanted to achieve, especially given attendees came from very different disciplines. A sense of ambition emerged from these early discussions, and a strong feeling that we could do something quite profound if we worked together across disciplines (cross-disciplinary working is revisited in later sessions). There was also a keen desire to make an impact, which led to a strong emphasis throughout on the end-to-end data pathway from capture to its eventual communication, and how to inform society and policy makers.

The initial activity on motivations was followed by a series of five short 5-min provocations by select attendees, selected for their ability to introduce more radical ideas into the ongoing conversation. These provocations were on the topics of:

• Self-organizing and self-adaptive systems in managing complexity (Ada Dionescu)
• Technology futures and the cross-disciplinary challenge (Rachel Prudden)
• Virtual labs of the future (Chantal Huijbers)
• From environmental statistics to environmental data science (Phil Jonathan)
• Everything EverywhAir: Measuring everything everywhere for air quality (Stefan Reiss)

The provocations were followed by a presentation and discussion on opportunities around the theme of the digital environment, led by Sophie Laurie from the Natural Environment Research Council in the UK. This presentation emphasized the timeliness of what we were discussing at the workshop and provided rich material to work with in subsequent sessions when we moved toward what we could achieve together.

Picking up on ambition, small groups were formed with the brief of working on how ambitious we could be. Important themes started to emerge at this stage, including the need to really grapple with uncertainty from a new, cross-disciplinary perspective; the importance of trust right the way through the chain of scientific discovery and decision making; and the need for new tools that will allow for increased representation of the complexities found in the natural environment, including tools that draw on studies of complexity.

The discussion then moved on to obstacles and barriers in order to make them explicit in our discussion. This identified issues such as the lack of incentives for cross-disciplinary, risky, and more long-term research; the lack of funding mechanisms and support structures to enable this; the challenges to a culture of open data and open science more generally; and the need to work within a system that emphasizes other issues, such as business innovation and growth. There was also strong recognition that there was a lack of trained people in this cross-disciplinary space.

The remainder of the workshop was then devoted to synthesizing the material and ideas into tangible outputs in terms of our desired vision and roadmap, and steps to building an international community. We were particularly seeking insights and outputs that could transcend the obstacles and barriers identified in the paragraph above. A panel of five people selected to be representative of the diversity in the summit was asked to distil the discussions into important elements of a roadmap. These were then discussed in depth by all attendees. This important process led to the emergence of the Windermere Accord, as presented below.

A parallel exercise, facilitated by artists in residence, was used to capture the personal stories and concerns of participants. This proved to be a core exercise, which brought the motivations/fears/aspirations of the participants right into the heart of the discussion. The exercise revolved around the following key questions: (1) what are your earliest formative experiences of nature? (2) What do you fear the next generation may not witness or experience in the natural world? (3) What can I/we do to address our disconnect with nature and better understand and manage the richness of environmental ecosystems? This culminated in a gallery around the room involving Polaroid images of all the participants and their statements in answer to these three questions. The collective responses have been distilled into a reflection,^19^ and also a poem reproduced in Appendix A.

Space was left during the workshop for group walks in nature and a boat trip, and these proved to be important in terms of enhancing dialogue and developing the conversations further in a more relaxed environment.

**Figure 1:**
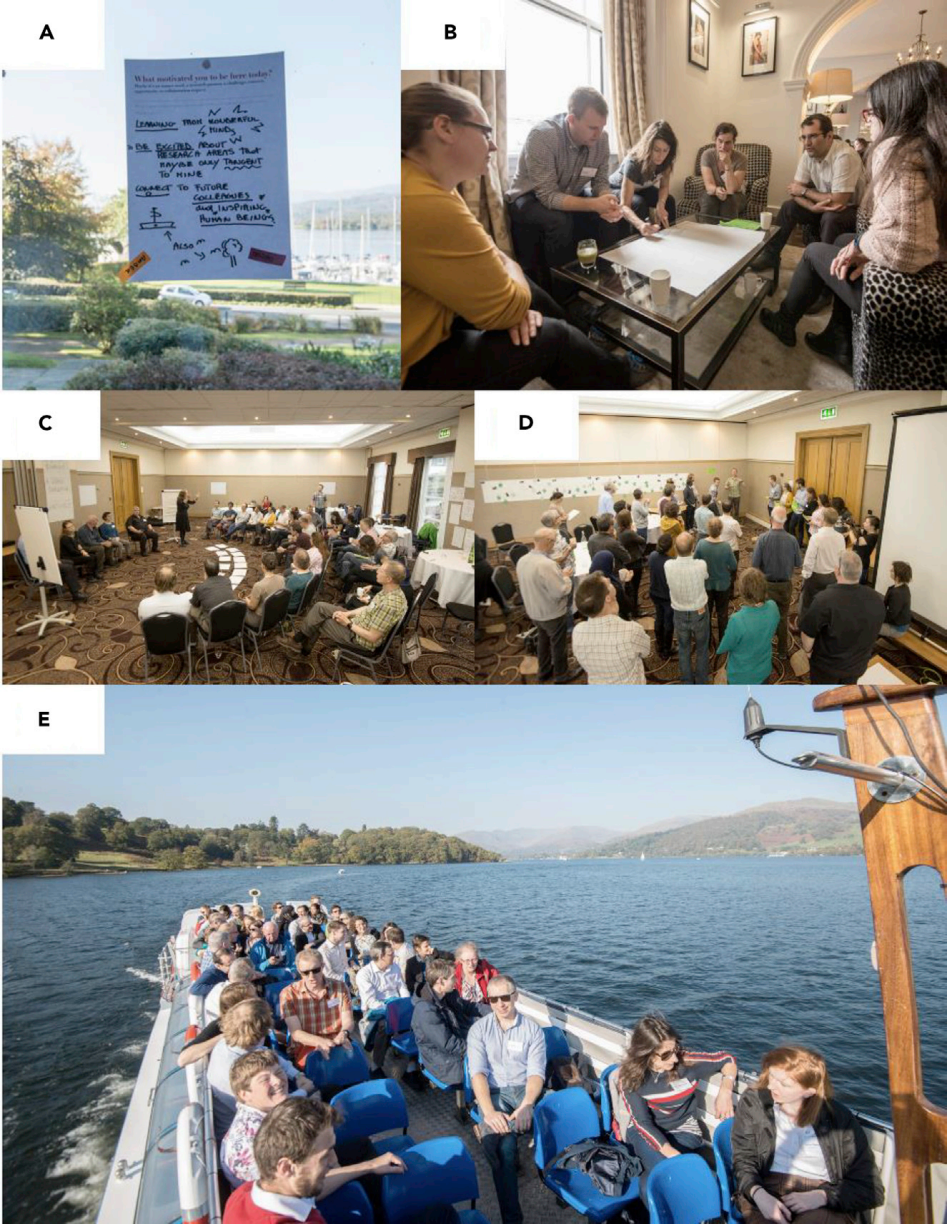
Images from the Summit (A−E) The initial trialogue session (A); small group working (B); synthesizing the outcomes (C); working with our artists in residence (D); relaxing and feeling inspired (E).

Images representing the different phases of the workshop can be found in Figure 1.

## The Windermere Accord

**Figure 2:**
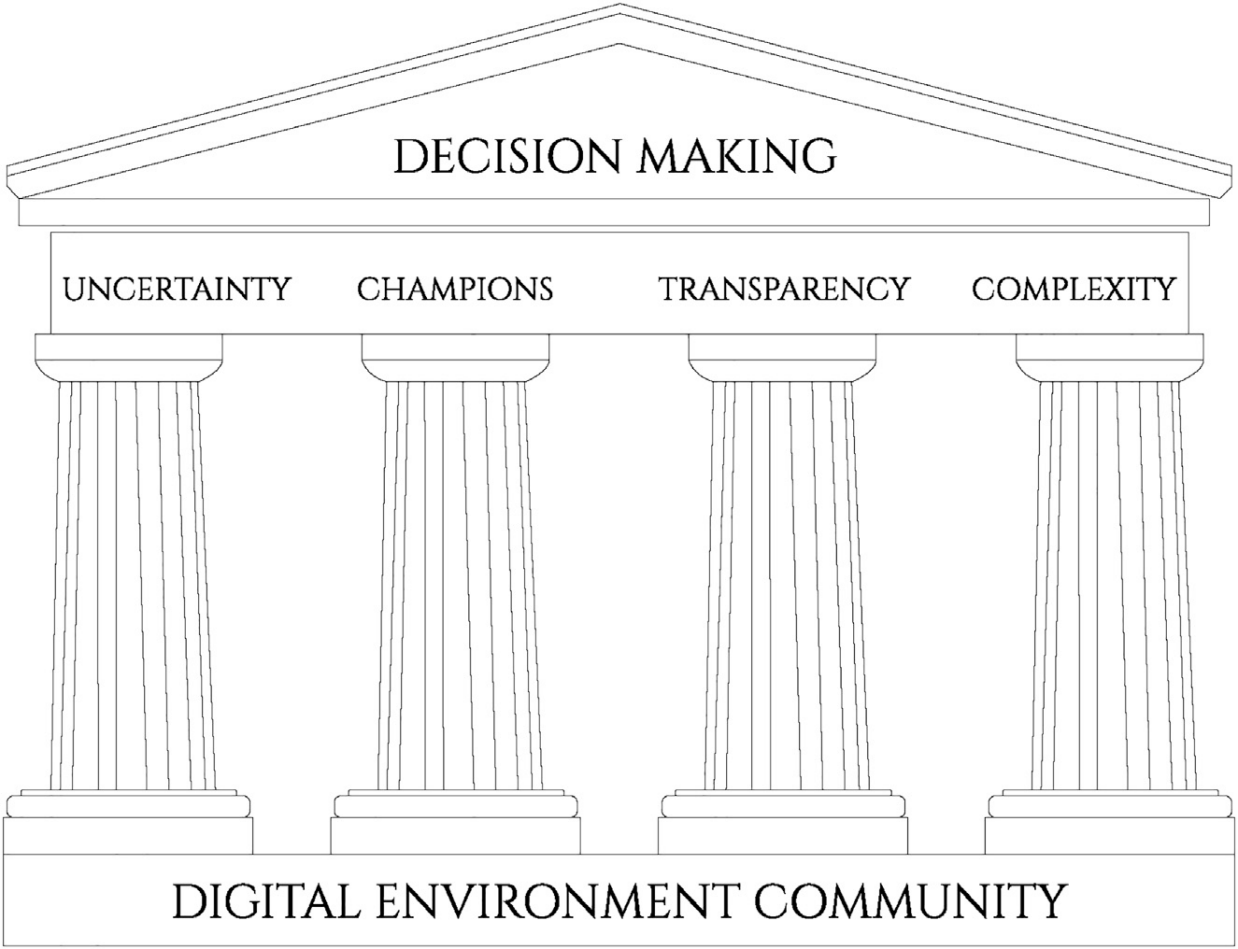
Pillars of the Windermere Accord

The summit produced a clear consensus over future directions around digital technology and the environment, resulting in what we refer to as the Windermere Accord, offering a framework and roadmap to take this area forward. This accord framework is depicted in Figure 2, with community as the base and four pillars all feeding into decision making (the archway).

The key elements of this framework are discussed in more detail below. The participants were also asked to state what they felt was most important to them for each element of the accord, and these were captured and are replicated in full in Appendix B, with key elements pulled out in the discussions below.

### Foundations: Building a Digital Environment Community

There was a strong consensus on the importance of building on the summit and developing a much larger international community working on the theme of a digital environment. There was a sense that the existing community is too small and fragmented and hence there is a need to make some noise about the importance of this area and also have a strong narrative around grand challenges in this area to draw others in (drawing on the work of champions as discussed in pillar 2). There is also a strong need to have mechanisms in place to support ongoing conversation on this topic, and to nurture and grow the community. In terms of concrete actions and next steps, the participants proposed creating integrating and fundamentally cross-disciplinary international conferences and journals in this area, and key to this is drawing together existing smaller communities such as Climate Informatics and ICT4S (see list presented earlier in this article). It is encouraging to see new journals emerging in this space. We also boldly propose a research institute around the digital environment (discussed further in next steps).

### Pillar 1: Using Technology to Tame Uncertainty

The first pillar focuses on uncertainty, particularly in how uncertainty can be estimated and managed in relation to environmental modeling.^20^ This is arguably the core challenge in supporting decision making in environmental science. Uncertainty may arise from a number of areas, including from the framing of the problem and consideration of external forces; data themselves and how they are measured; from the assumptions and structures within a given environmental model or models; from the parameter selection for that model; from how a model is implemented; and how results are analyzed, presented, and interpreted. This becomes a huge challenge when modeling complex systems involving model chains where results of one model feed into another model or models and where feedbacks need to be considered. Often uncertainty is considered from a statistical perspective. There was a consensus in the summit from our discussions that we need fresh perspectives on uncertainty. In particular, we need a cross-disciplinary approach to the subject taking input from statistics, data science, computer science, environmental sciences, social science, and arts-based subjects. It is also important that uncertainty is addressed in an end-to-end fashion from data acquisition through to visualizing and presenting uncertainty in support of decision making. Finally, place-based approaches are important, supported by rich data about that place (cf. the models-of-everywhere approach, which advocates collecting rich and varied environmental data about specific geographical locations to enhance knowledge about that particular place in all its dimensions^21^, ^22^, ^23^).

### Pillar 2: Advocates and Champions to Enable, Empower, and Influence

The second pillar focuses on people and, in particular, identifying and developing a generation of leaders to take forward the rich agenda on the digital environment. We identified the importance of having people who understand both the capabilities of digital technologies and also the challenges of the environmental sciences, seeing such “glue people” as crucial in the development of this area. We also recognize that such people are in scarce supply so additional training is urgently needed. Furthermore, there is a need to raise the profile of environmental challenges to draw people toward this field, especially given the financial rewards of taking their digital skills elsewhere. This includes communicating scientific questions and challenges and their significance. A number of the attendees also asserted that we can all be champions, taking leadership in this area now and helping it to thrive.

### Pillar 3: Digital Technology Leading the Way in Openness and Transparency

There was strong recognition that contemporary digital technologies enable a new kind of science that is open, transparent, and also completely reproducible, and this is also essential in terms of enhancing trust. Participants also highlighted the importance of honesty and full disclosure of scientific limitations in enhancing this trust. We see cloud computing as crucial in providing the core building block to support this openness and transparency, especially when coupled with the scalability inherent in cloud technologies. This is greatly enhanced by virtual labs offering integrated data, modeling, and analyses around a particular (collaborative) scientific quest.^24^ It is also important that audit trails can be provided, and, again, recent technological advances can support this (e.g., blockchain technology^25^). While this is now technically feasible, there was recognition that there has to be a strong cultural shift toward openness across the community.^7^

### Pillar 4: Integration and Feedbacks in Complex Systems

Environmental systems are highly complex systems and scientists need new tools to understand this complexity.^26^^,^^27^ There was a high level of agreement in the summit that digital technology can provide a new set of tools to enhance our understanding of this complexity in terms of supporting a more holistic approach to science inspired by systems thinking. This includes the development of software frameworks to support integrated environmental modeling around ecosystem services, including more sophisticated support for model coupling and also enhanced techniques to understand feedbacks in such integrated systems. We note existing studies that argue for the benefits of advanced software engineering principles and techniques in support of sustainability research, particularly in managing complexity.^28^ There was also recognition of the potential role of autonomic computing^29^^,^^30^ in managing this complexity and also supporting reasoning across scales, complementing existing approaches based on data assimilation.^31^^,^^32^ Can knowledge gained from data analyses be used to more precisely dynamically define model parameterization to ensure that models represent current observations? Going further, is it possible, for example, for environmental models to self-organize or adapt their fine-grained behavior to match observations over time? Can measures of uncertainty in models be used to determine adaptive sampling strategies to generate the necessary additional data to reduce such uncertainties? As with uncertainty, the key message is that it is timely to re-examine complexity from a fresh, cross-disciplinary perspective.

### Archway: Decision Making

The final part of the accord was recognition that the various pillars and the underpinning community are all mechanisms to support more informed decision making and indeed this is core to everything we do around a digital environment. There is a tremendous opportunity to develop decision-support systems based on rich environmental data, and this requires innovations at each step of the chain from data acquisition through to the presentation of the analyses. These various steps need to be brought together in one logical place, hence our emphasis on virtual labs in pillar 3, which we now say should offer explicit support for decision making. We see a strong role for creative data visualization and presentation, and this again needs a cross-disciplinary approach requiring input from arts disciplines. There was also recognition that this support is required across all scales from individual decisions, through local decision making, to regional, national, and global decisions around environmental change. This relates strongly to the goal of translating data to information to knowledge and eventually to wisdom, a stated motivation behind AI.

**Figure 3:**
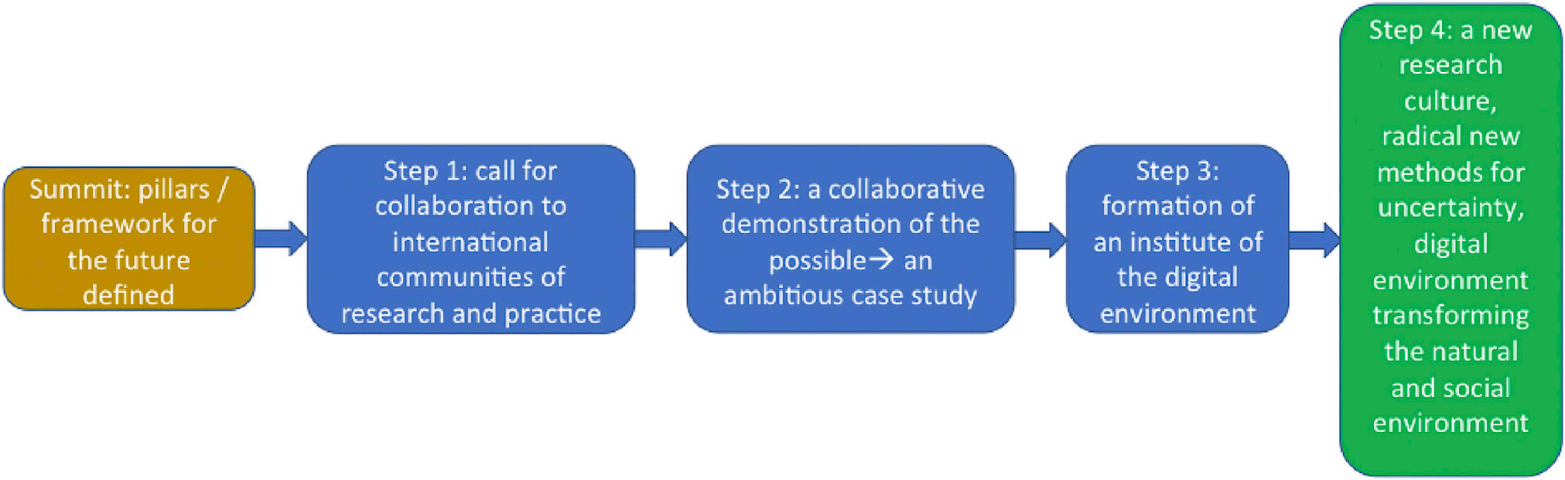
The Associated Roadmap

The summit concluded with a proposed roadmap in the form of a series of next steps leading to a new cross-disciplinary research culture informed by further work on the different pillars (Figure 3). Note that these steps are also not necessarily sequential and would be more agile and overlapping in practice. The summit is a small step toward such a vision, and the authors, as the participants in this summit, pledge to embrace this new culture and now reach out to others to join together in this quest for a new data-enriched, collaborative approach to some of the biggest grand challenges of our time.

Since the summit, quite a lot has changed, including increasing motivation and promising initial steps toward our vision. If anything, climate change is even more in focus having witnessed the Australian bushfires and extensive floods and droughts worldwide, and increasing voices for change often inspired by Greta Thunberg. The current coronavirus disease 2019 (COVID-19) pandemic has also been linked to interference in nature. We are also seeing growing interest in the role of digital technology in the environment. In the UK context, there have been considerable developments within the CDE program introduced earlier, with a series of pilot projects now up and running and larger demonstrator projects about to be awarded. This level of research and innovation activity is also reflected in other countries. For example, in Australia we see significant investment in digital platforms for climate research (e.g., the Ecocommons program^33^). We also see international initiatives particularly around technological platforms, including the European Open Science Cloud,^34^ D4Science,^35^ and Pangeo.^36^

Returning to CDE, it is interesting to note that the program very quickly took three complementary actions: (1) it appointed champions for the program; (2) it set up the Digital Environment Expert Network, which also includes early career researchers (again representing a concrete step to broaden the number and range of champions); and (3) it recognized the importance of cross-disciplinary thinking through the multi-disciplinary and interdisciplinary research and innovation (MIDRI) initiative that sits at the heart of the program. There is also an emphasis on demonstrators in this program (cf. case studies as identified in step 3 of our pathway). These are important steps that are very much in line with the accord. In a UK context, this is also a model that could be replicated elsewhere. The publication of this perspective also represents an important call for collaboration (step 1 of Figure 3). Internationally, there are other interesting developments but the position is still rather fragmented, so it is timely to repeat our call to draw together internationally to create a strong cross-disciplinary community to work on this urgent and important topic. It would be fantastic to see a truly global Institute of the Digital Environment emerge in the post-COVID-19 world, pushing from progress on steps 1 and 2 toward the latter stages of our roadmap.

## Concluding Remarks

This short paper has presented the outcomes of a summit on the role of digital technology in responding to the grand challenges of environmental change, a unique cross-disciplinary gathering bringing together environmental scientists, data scientists, computer scientists, social scientists, and the representatives of the creative arts. The key output of this workshop was an agreement of a vision and framework/roadmap for this important area, captured in the Windermere Accord. This accord envisions a new kind of environmental science underpinned by unprecedented amounts of data, with technological advances leading to breakthroughs in taming uncertainty and complexity, and also supporting openness, transparency, and reproducibility in science. These are precisely the tools that are required by decision makers at all levels to make more well-informed decisions in the face of profound environmental change. Crucially, though, to support this it is essential to build a cross-disciplinary community working on these themes and also to identify and grow champions for this area.

## Supplemental Information

Document S1

Document S2. Article plus Supplemental Information
