# Supplementary material for: The Role of Digital Technologies in Responding to the Grand Challenges of the Natural Environment: The Windermere Accord
Source: Patterns (N Y). 2021 Jan 8;2(1):100156. doi: 10.1016/j.patter.2020.100156 (PMC7815947; doi:10.1016/j.patter.2020.100156)
Supplement: Document S1 [file mmc1.pdf]

PATTER, Volume *2*

Supplemental Information

The Role of Digital Technologies in Responding to the Grand Challenges of the Natural Environment: The Windermere Accord

Gordon S. Blair, Richard Bassett, Lucy Bastin, Lindsay Beevers, Maribel Isabel Borrajo, Mike Brown, Sarah L. Dance, Ada Dionescu, Liz Edwards, Maria Angela Ferrario, Rob Fraser, Harriet Fraser, Simon Gardner, Peter Henrys, Tony Hey, Stuart Homann, Chantal Huijbers, James Hutchison, Phil Jonathan, Rob Lamb, Sophie Laurie, Amber Leeson, David Leslie, Malcolm McMillan, Vatsala Nundloll, Oluwole Oyebamiji, Jordan Phillipson, Vicky Pope, Rachel Prudden, Stefan Reis, Maria Salama, Faiza Samreen, Dino Sejdinovic, Will Simm, Roger Street, Lauren Thornton, Ross Towe, Joshua Vande Hey, Massimo Vieno, Joanne Waller, and John Watkins

Appendix A: What comes next?

Inspired by the Windermere Accord Summit, October 2018
Harriet Fraser

Estimated anthropogenic global warming is currently increasing at 0.2°C (likely between 0.1°C and 0.3°C) per decade due to past and ongoing emissions (high confidence).

Where do you want to walk?
Shall we stroll together through the Valley of Despair,
kick our feet through leaves of fear,
brush against obstacles in industry, academia, politics?
Shall we wander in this fog endlessly?
Shall we retreat?

Avoiding overshoot and reliance on future largescale deployment of carbon dioxide removal (CDR) can only be achieved if global CO2 emissions start to decline well before 2030 (high confidence).

Or shall we stride up to the heights
to feel the light and take a wider view?
Shall we be ambitious, and push modesty away,
negotiate obstacles, face uncertainty,
keep our feet on the ground, earthed,
and as a community propose a new road map?

With 1.5°C of global warming, one sea ice-free Arctic summer is projected per century. This likelihood is increased to at least one per decade with 2°C global warming.

We have seen coral reefs dying
We have imagined their passing
We have modelled their death

We have imagined summer fields without butterflies
We have watched decline
We have modelled depletion
We have imagined a world without wild

The risk of irreversible loss of many marine and coastal ecosystems increases with global warming, especially at 2°C or more (high confidence).

What else might we imagine
from the sunlit heights?
Can we imagine a rapid end
to the toxic emission of carbon dioxide,
to the warming?
Can we imagine forests, growing

or the ocean, clean?
Can we make that happen?

Education, information, and community approaches, including those that are informed by Indigenous knowledge and local knowledge, can accelerate the wide scale behaviour changes consistent with adapting to and limiting global warming to 1.5°C.

There's pressure
to look for what you expect to see
where you expect to see it
but that's not where the interesting stuff lies

There are always questions
Where do we predict that we are going?
What do we need to know?
If we use models as tools to think with
are we choosing the right models?

Limiting global warming to 1.5°C, compared with 2°C, could reduce the number of people both exposed to climate-related risks and susceptible to poverty by up to several hundred million by 2050 (medium confidence).

Who's in the community?
What's in our tool box?
Sensors, computers, hope,
Knowledge, stories, reason
Empathy, connection, drive

We are part of the system:
systems within systems,
creating, adapting, imagining, learning
in the human-digital age,
integrated, inter-connected,
as natural systems are,

and we know: *Collective efforts at all levels,*
in ways that reflect different circumstances and capabilities,
in the pursuit of limiting global warming to 1.5^{o}C,
taking into account equity as well as effectiveness,
can facilitate strengthening the global response to climate change,
achieving sustainable development
and eradicating poverty
(high confidence).

Quotes taken from the IPCC (Intergovernmental Panel on Climate Change) report summary for Policy Makers, October 2018

Appendix B: Annotations around the elements of the Accord

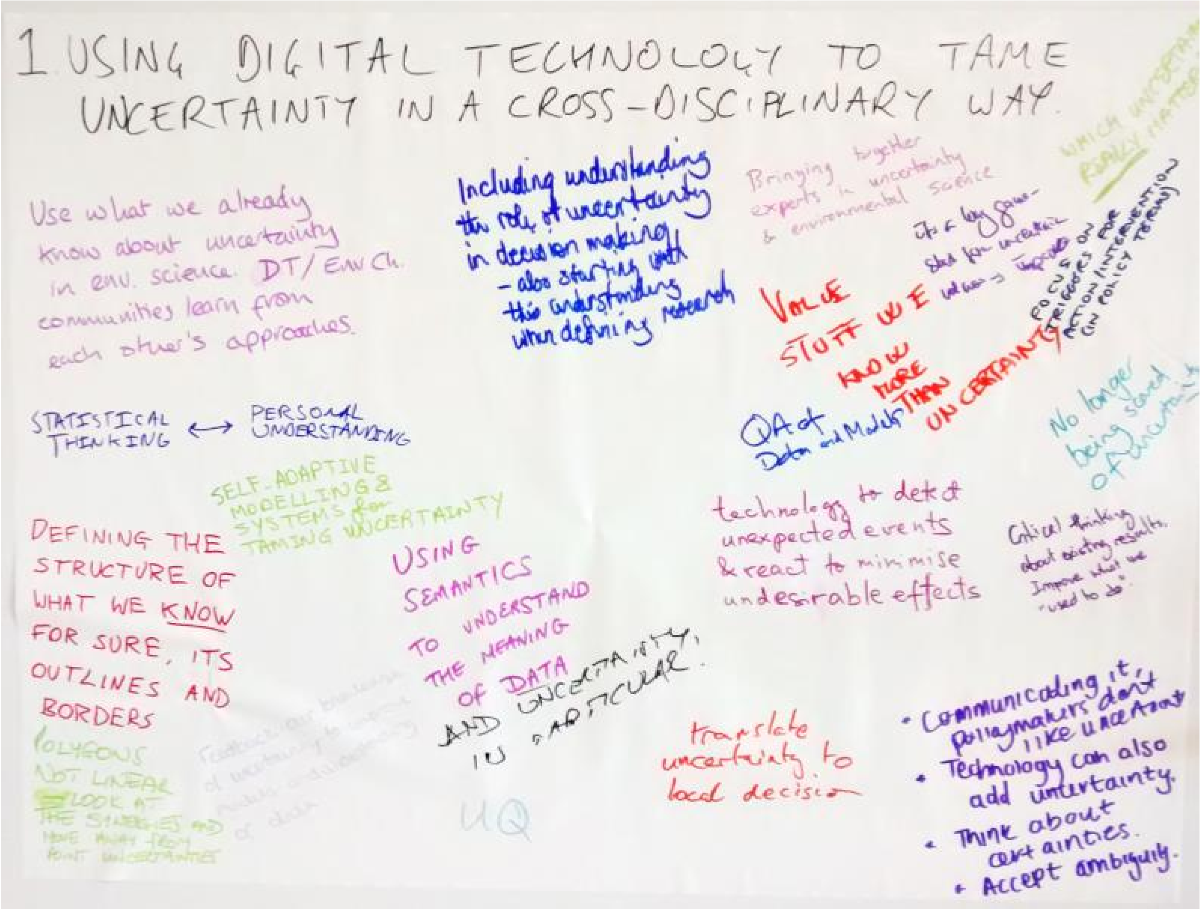

Extract meaning from uncertainty in decision contexts

don't look to fully close uncertainty gap just improve it.

SPECIFIC CHALLENGES & COMMON LANGUAGE

Need to 'anchor' these abstract notions in concrete environmental context. Can one have an equivalent of "1000 genomes project" for example? How about "The Windermere project" capturing environmental change data over 50 – 100 yrs?

Capture Uncertainty which is comming from digital technology itself.

1

Communicating uncertainty not just in stats but in visual ways, "show & tell" evidence

a virtual catchment ⇒ where everything complex nat'ls and all are integrated (feedback + complexity) is modelled & explored

⇒ Can we embrace uncertainty instead of taming? Then understand where we need more knowledge/process/complexity in order to effect/influence decisions? Complex into concrete? ↑ help messaging

2. ADVOCATES + CHAMPIONS TO ENABLE, EMPOWER AND INFLUENCE

Training

Need for training next generation leaders in effective comm^n methods

Use every opportunity to spread the word responsibly

How do we build trust across gaps

EMPATHY

give a voice to those who do not (feel they) have one

LISTEN!!! don't just tell

THINK OF WHAT IS AT STAKE

Effective two-way KE and translation

Training at all levels
- studentships
- senior academics (cross-disciplinary/ tech term translation)
- language
- business/policymaker facing
- people (general public)
- children

EVERYONE OF US IS A CHAMPION & INFLUENCER

CAN'T RELY ON SMALL NUMBER OF INDIVIDUALS

CRITICAL THINKING AND AUTONOMY

BIG DATA ANALYSIS TO CREATE MORE INFLUENCING OPPORTUNITIES

Identity + encourage them – who can!

We can all be champions, we can all have give, we don't need to look for specific grants to stand on the shoulders of.

Structures to assist this.

help identify responsibility

ENGAGE WITH PUBLISHED POLICY TO ANTICIPATE EVIDENCE NEEDS E.G.:
- DEFRA 25 YEAR ENV. PLAN
- WALES ENVIRONMENT ACT + WELLBEING OF FUTURE GENERATIONS ACT

LET EXPERIENCE SPEAK

GLUE MAKERS/ENABLERS ARE ACCOUNTABLE AND TRUSTED

Modest leaders in digital domains have to play their role as champions

MAKE "AI FOR ENVIRONMENT" THE NEXT ALPHAGO MOMENT

CREATE FORA WHERE ADVOCATES CAN EXPRESS KNOWLEDGE NEEDS TO DIRECT RESEARCH AND CHAMPIONS CAN SHARE RESEARCH TO INFORM DECISION MAKING

USE ADVOCATES FOR INFLUENCING KEY GOVT. DECISIONS.
USE CHAMPIONS TO DEVELOP THE NETWORK/ COMMUNITY (& THE EMERGING FIELD)

Identify & train the glue people → who are these glue people??

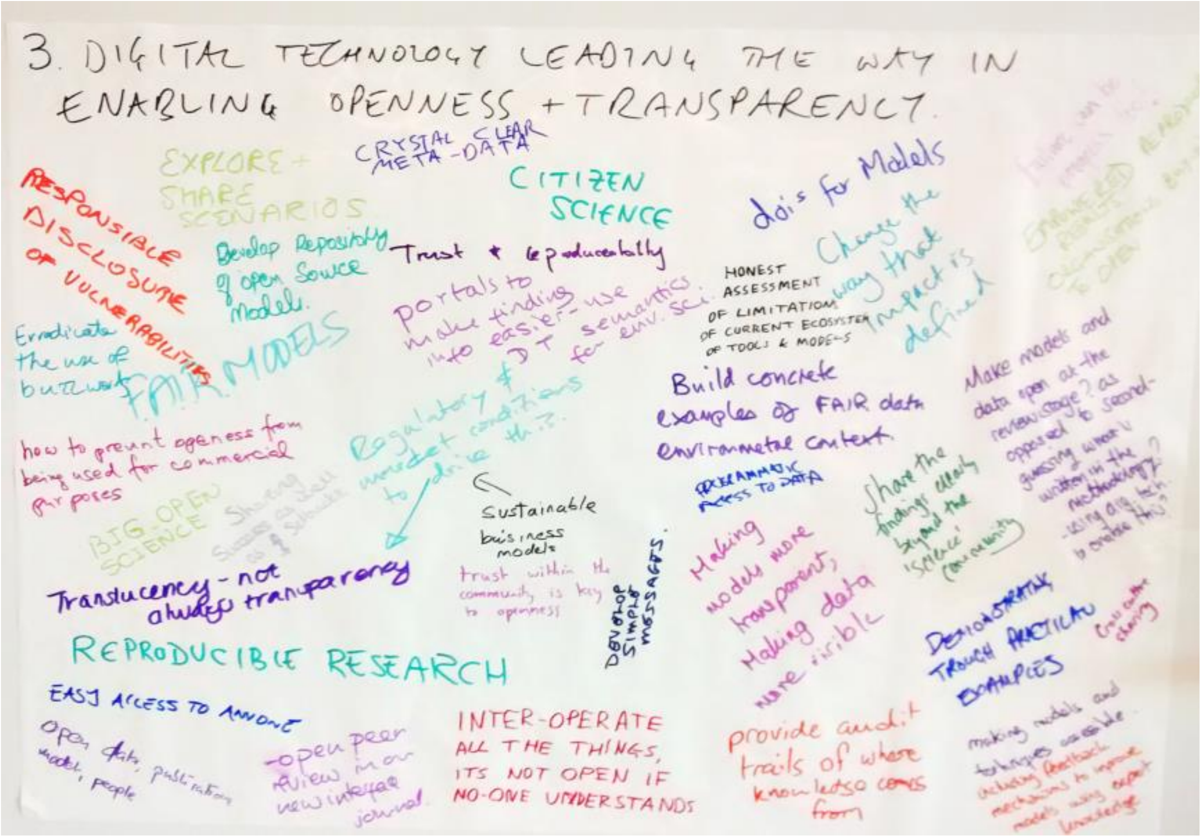
3. DIGITAL TECHNOLOGY LEADING THE WAY IN ENABLING OPENNESS + TRANSPARENCY.
CRYSTAL CLEAR META-DATA
EXPLORE + SHARE SCENARIOS
CITIZEN SCIENCE
dois for Models
RESPONSIBLE DISCLOSURE OF VULNERABILITIES
Develop Repository of open Source models.
Trust + reproducibility
Change the way that impact is defined
HONEST ASSESSMENT OF LIMITATION OF CURRENT ECOSYSTEM OF TOOLS & MODELS
portals to make finding easier - use semantics for env. sci. into DT
Eradicate the use of buzzwords
FAIR MODELS
Build concrete examples of FAIR data environmetal context
Make models and data open at the review stage? as opposed to second-guessing what's written in the methodology? - using dig. tech. to enable this?
how to prevent openess from being used for commercial purposes
Regulatory & market conditions to drive this?
Sustainable business models
PROGRAMMATIC ACCESS TO DATA
Share the findings clearly beyond the 'science' community
BIG OPEN SCIENCE
Translucency - not always transparency
trust within the community is key to openness
DEVELOP SIMPLE MESSAGES.
Making models more transparent, Making data more visible
DEMONSTRATING THROUGH PRACTICAL EXAMPLES
REPRODUCIBLE RESEARCH
EASY ACCESS TO ANYONE
Open data, publication, model, people
open peer review in our new interface journal
INTER-OPERATE ALL THE THINGS, ITS NOT OPEN IF NO-ONE UNDERSTANDS
provide audit trails of where knowledge comes from
making models and techniques accessible
including feedback mechanisms to improve models using expert knowledge

4. INTEGRATION AND FEEDBACK

(WE KNOW WHAT WE MEAN)

multi-scale self-adaptive integration

A good example helps... Can we try to do it for somewhere.

Integrative solution oriented tools & methods

Collaborative working practice to bring approaches together

Knowledge map - gap analysis - big env. Qs
- what can be done already?
- what needs improving?
- which methods (data sci/physical process) are better when?

Tolerance and understanding of different perspectives.

Brand as "AI for environmental Science" for political relevance

↑ we shouldn't pander to the terms 'of the day' we should hold ourselves in higher esteem?

Semantics + uncertainty within semantic modelling

DYNAMIC ADAPTIVE MODELS & SYSTEMS

PROVIDE AN INTEGRATED VIEW (NOT THE SAME AS HOMOGENEOUS)

FOCUS ON WHAT MAKES A DIFFERENCE AND MAKE COMPLEX PROBLEMS TRACTABLE (80/20 IT!)

Technology for Adaptive integration

Falicitating the communication between different scientific disciplines

Linking platforms to enable cross-disciplinary and whole of data life cycle research approach.

Environmental Equilibrium in totality.

Being able to understand how your work fits into the bigger picture

avoiding trade-offs not being accounted for and sub-optimal decisions leading to worse off situation

20% PROXIMITY!

NEED TO BE AGILE + ADAPTIVE

Just do it? and don't worry about it failing?

Social narratives + social sciences to add richness + stories to data & tech

MULTIMODAL DATA

INTEGRATED PLURALITY OF VIEWPOINTS

"NOT JUST PASSIVE [illegible] - no skinny [illegible]"

ENABLE SYSTEMS TO SELF LEARN TO INFORM OPERATORS/ USERS/DECISION-MAKERS

Developing a common language to facilitate the integration

NEW WAYS OF TACKLING COMPLEX PROBLEMS WITH DISTRIBUTED TEAMS AND SHARING KNOWLEDGE RAPIDLY ACROSS TEAMS GLOBALLY

Self Adaptive models with a feedback loop ~~some supporting framework~~

Using digitalisation of the environment & data science techniques to better understand climate feedback mechanisms

IMPACTS OF MULTIPLE DRIVERS ON MULTIPLE ENDPOINTS

never stop learning and allowing for change.

4

Need to plan feedbacks into project lifecycles, otherwise information may be lost after 3/5 years when projects finish.

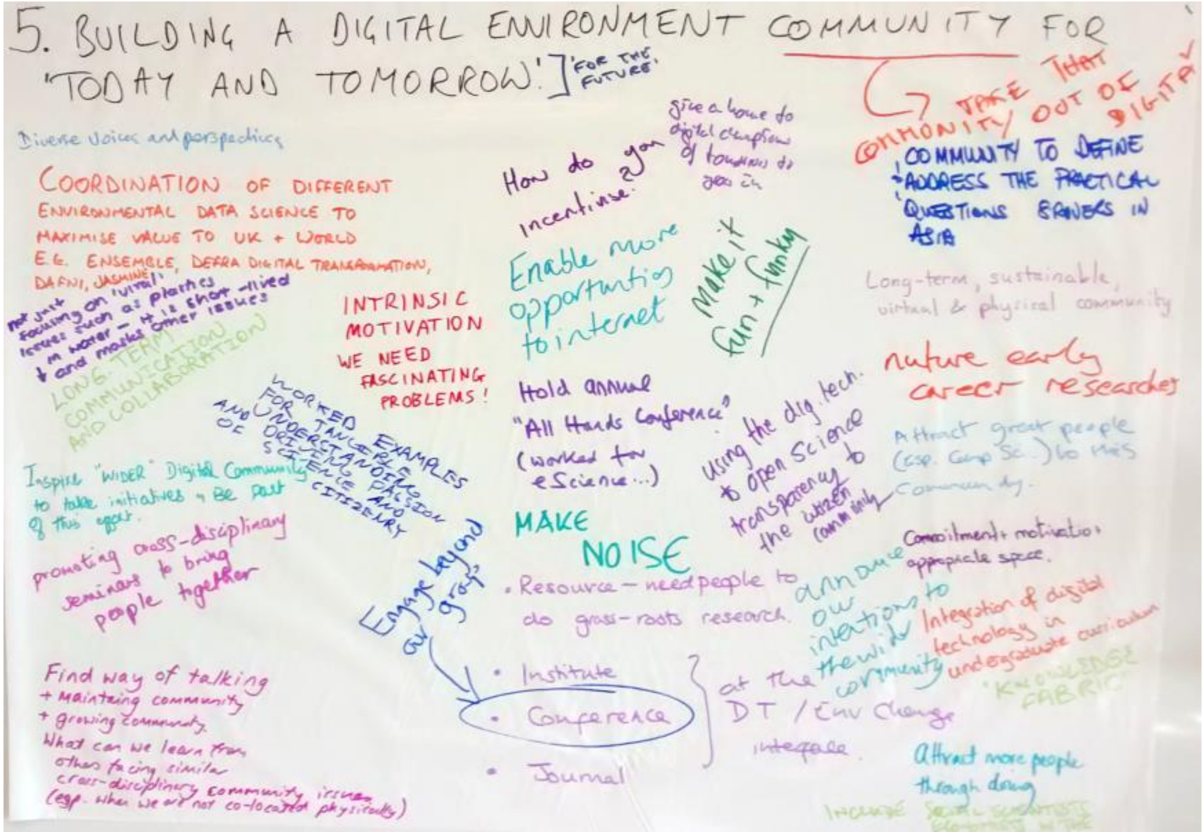
5. BUILDING A DIGITAL ENVIRONMENT COMMUNITY FOR
'TODAY AND TOMORROW'.] 'FOR THE FUTURE'
Diverse voices and perspectives
COORDINATION OF DIFFERENT ENVIRONMENTAL DATA SCIENCE TO MAXIMISE VALUE TO UK + WORLD
E.G. ENSEMBLE, DEFRA DIGITAL TRANSFORMATION, DAFNI, JASMINE
not just focusing on 'viral' issues such as plastics in water – it is short-lived & and masks other issues
LONG-TERM COMMUNICATION AND COLLABORATION
INTRINSIC MOTIVATION
WE NEED FASCINATING PROBLEMS!
WORKED EXAMPLES FOR TANGIBLE UNDERSTANDING AND DRIVING PASSION OF SCIENCE AND CITIZENRY
Inspire "WIDER" Digital Community to take initiatives & be part of this effort.
promoting cross-disciplinary seminars to bring people together
Find way of talking + maintaining community + growing community.
What can we learn from others facing similar cross-disciplinary community issues (esp. when we are not co-located physically)
How do you incentivise?
give a home to digital champions of tomorrow to grow in
Enable more opportunities to internet
Make it fun + funky
Hold annual "All Hands Conference" (worked for eScience...)
MAKE NOISE
Engage beyond our group
• Resource – need people to do grass-roots research.
• Institute
• Conference
• Journal
at the DT / Env Change interface.
Using the dig. tech. to open science & transparency to the wider community
announce our intentions to the wider community
TAKE THAT COMMUNITY OUT OF DIGITAL
COMMUNITY TO DEFINE & ADDRESS THE PRACTICAL QUESTIONS & ANSWERS IN ASIA
Long-term, sustainable, virtual & physical community
nurture early career researchers
Attract great people (esp. Comp Sc.) to this community.
Commitment + motivation appropriate space.
Integration of digital technology in undergraduate curriculum
'KNOWLEDGE FABRIC'
attract more people through doing

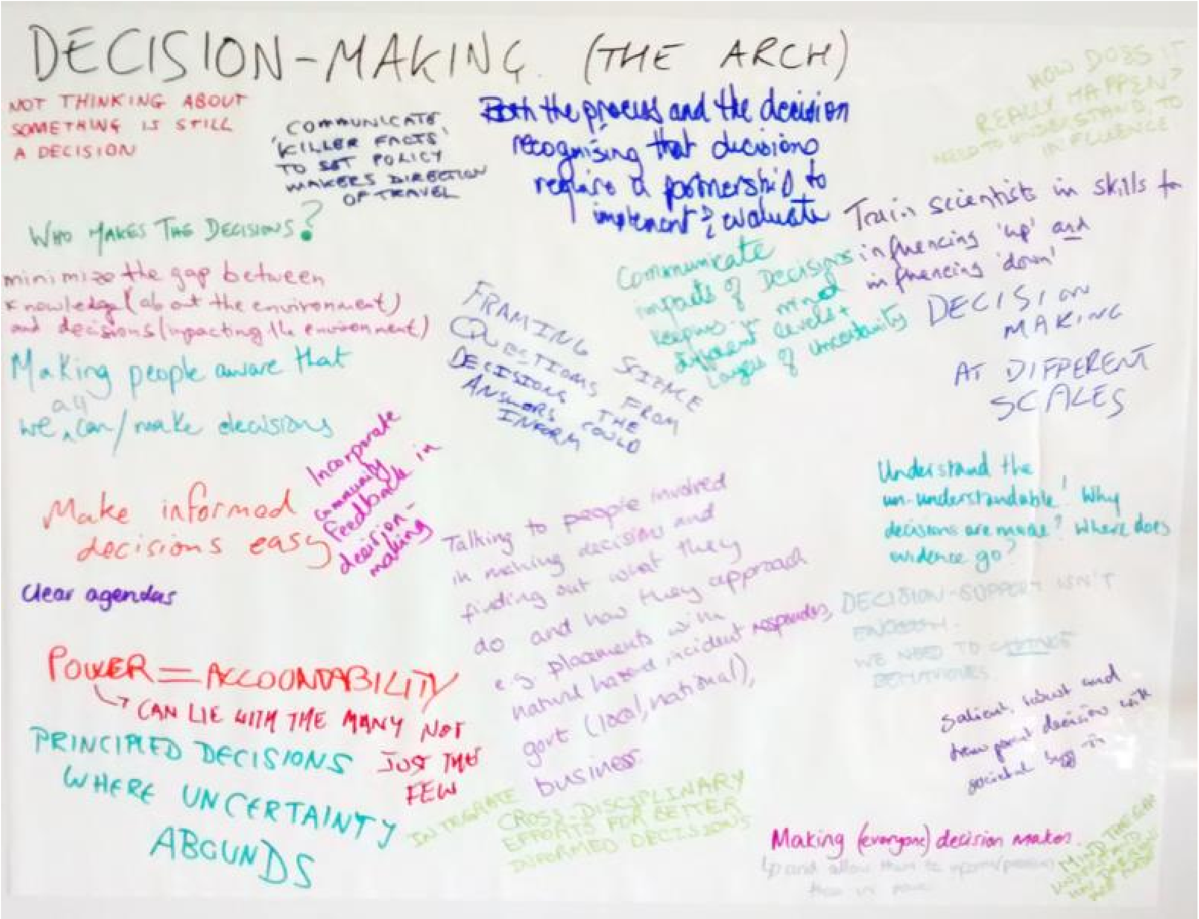
DECISION-MAKING (THE ARCH)
NOT THINKING ABOUT SOMETHING IS STILL A DECISION
COMMUNICATE 'KILLER FACTS' TO SET POLICY MAKERS DIRECTION OF TRAVEL
Both the process and the decision recognising that decisions require a partnership to implement & evaluate
HOW DOES IT REALLY HAPPEN? NEED TO UNDERSTAND, TO INFLUENCE
WHO MAKES THE DECISIONS?
Train scientists in skills for influencing 'up' and influencing 'down'
Communicate impacts of Decisions keeping in mind different levels + layers of uncertainty
minimize the gap between knowledge (about the environment) and decisions (impacting the environment)
FRAMING SCIENCE FROM QUESTIONS THE DECISIONS COULD ANSWERS INFORM
DECISION MAKING AT DIFFERENT SCALES
Making people aware that we all can/make decisions
Make informed decisions easy
Incorporate community feedback in decision-making
Understand the 'un-understandable'. Why decisions are made? Where does evidence go?
Talking to people involved in making decisions and finding out what they do and how they approach e.g. placements with natural hazard incident responders, govt (local, national), business
Clear agendas
POWER = ACCOUNTABILITY
CAN LIE WITH THE MANY NOT JUST THE FEW
PRINCIPLED DECISIONS WHERE UNCERTAINTY ABOUNDS
INTEGRATE CROSS-DISCIPLINARY EFFORTS FOR BETTER INFORMED DECISIONS
Salient, robust and transparent decisions with societal buy in
Making (everyone) decision makers
